# The intrinsic reason why it is relevant to introduce the concept of “small fiber neuralgia” into the taxonomy of pain disorders

**DOI:** 10.3389/fpain.2026.1820678

**Published:** 2026-04-13

**Authors:** Jean-Pascal Lefaucheur

**Affiliations:** 1 UR4391 (ENT Team), Faculty of Health, Paris Est Créteil University, Créteil, France; 2 Department of Clinical Neurophysiology, Henri Mondor University Hospital, AP-HP, Créteil, France

**Keywords:** hyperexcitability, neuralgia, neuropathic pain, peripheral neuropathy, small nerve fibers

The term “neuralgia” refers to various painful conditions involving a nerve trunk, such as trigeminal neuralgia, occipital neuralgia, glossopharyngeal neuralgia, intercostal neuralgia, or pudendal neuralgia. In these conditions, the pain is essentially linked to a phenomenon of mechanical nerve compression by a blood vessel, a bone structure or a ligament. Nerve compression preferentially affects large-diameter nerve fibers (Aβ fibers) rather than small-diameter ones (1). This is why the pain is expressed as a sensation of electric shock rather than burning, for example. In fact, compression initially causes hyperexcitability of the Aβ fibers which become abnormally hyperactive with high-frequency ectopic discharges at the origin of paresthesia sensations (2). In more advanced processes, this can be associated with structural damage to impinged or entrapped Aβ fibers, including demyelination, which also promotes axonal hyperexcitability (3), but such damage is not systematic. Indeed, situations of pure hyperexcitability exist, particularly in the context of vascular conflict on the cranial nerves causing neuralgia. Neurovascular conflicts are generally located at the nerve root entry zone, in a transition zone between central and peripheral myelin (4), where the epineurium and the interfascicular connective tissue (perineurium) separating the fibers are absent, thus promoting hyperexcitability and ephaptic phenomena. Sensory symptoms related to cranial nerve hyperexcitability nearly always disappear immediately following a surgical procedure of microvascular decompression, providing formal evidence of a “functional” nerve excitability disorder underlying the clinical presentation of neuralgia (5, 6). When the lesion phenomenon is more pronounced, with a proven loss of large nerve fibers, the diagnosis becomes that of a “painful neuropathy” rather than “neuralgia”.

Small-diameter sensory nerve fibers (A*δ* and C fibers) can also obviously be involved in peripheral neuropathic pain, especially since nociceptive information is carried by these small fibers and not by large Aβ fibers in physiological conditions. In pathological conditions, both large- and small-diameter nerve fibers can exhibit abnormal hyperexcitability/hyperactivity leading to pain, but due to different causal mechanisms. While large fibers are more often involved in situations of mechanical compression, as mentioned previously, small fibers are more sensitive to inflammation or ischemia (7, 8). As for large fibers, small-fiber-dependent pain primarily relies on axonal hyperactivity, whether or not associated with structural nerve fiber damage (demyelination or axonal degeneration or loss). Therefore, two conditions can theoretically be distinguished: “small fiber neuralgia”, characterized by neuropathic pain involving abnormal hyperactivity of small fibers but without structural nerve fiber damage, and “painful small fiber neuropathy”, in cases of associated structural nerve fiber damage. This distinction may seem formal, but it is actually very important in clinical practice. According to the current algorithms (9, 10), the “definite” diagnosis of neuropathic pain relies on demonstrating a lesion of the nervous system through complementary tests. In this case, neuropathic pain could be ruled out in the absence of a proven lesion and objective abnormalities on examination. However, it is clear that neuropathic pain can exist in a situation of pure nerve hyperexcitability, without structural damage, which highlights the limitations of complementary tests in the diagnosis of neuropathic pain (11).

The existence of neuropathic pain involving small fibers in the absence of structural lesions of these fibers has been clearly demonstrated by the discovery of gain-of-function mutations in genes encoding skin nociceptors (*TRPA1*) (12) or sodium channels of axonal membrane (*SCN9A-10A-11A*) (13–15), which are involved in the propagation of action potentials along small diameter nociceptive nerve fibers. For example, a gain-of-function mutation in *TRPA1* causes severe paroxysmal pain without any reduction in intraepidermal fiber density (12). More generally, spontaneous neuropathic pain may primarily involve hyperexcitability of small fibers (16), as shown by many cases of erythromelalgia, which can occur without loss of small fibers in the extremities (17, 18). These more or less focal situations of small fiber hyperexcitability/hyperactivity can be described as “neuralgic” rather than “neuropathic”, in the absence of structural axonal lesion.

**Figure 1:**
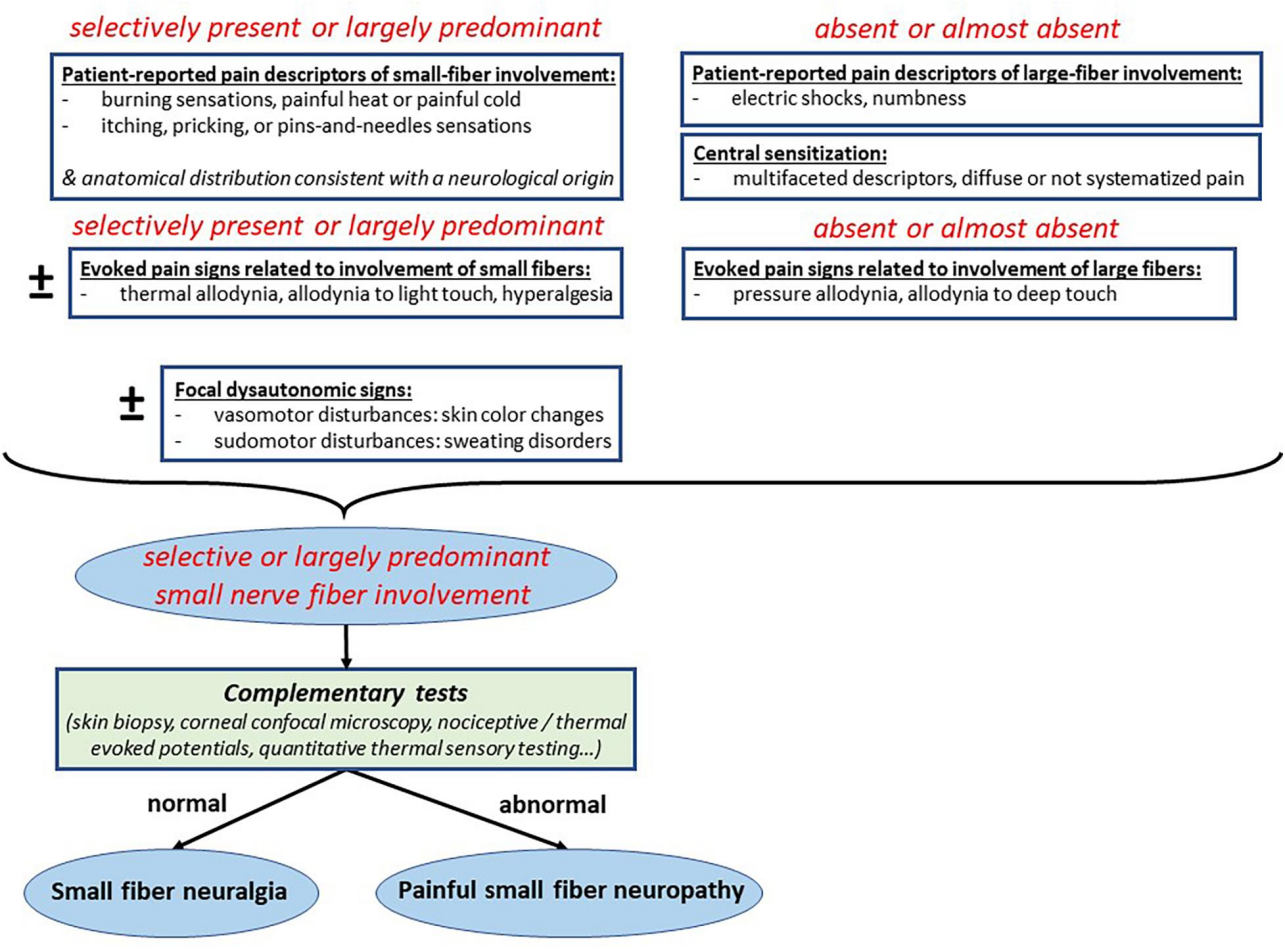
Proposed algorithm for the differential diagnosis between small fiber neuralgia and painful small fiber neuropathy.

Thus, clinical examples of large fiber neuralgia are well-defined, such as classical trigeminal neuralgia (19). On the other hand, cases of neuralgic phenomena, that is to say occurring without structural lesions leading to a loss of fibers, and selectively affecting small fibers, are less well characterized, but they do exist. The selective involvement of small fibers in a chronic pain condition can only be clinically defined by “qualitative clinical characteristics” in the absence of questionnaires exhibiting such specificity of neuropathic involvement (20). These “qualitative clinical characteristics”, detailed in Figure 1 include (i) specific spontaneous pain descriptors, such as burning sensations, painful heat or painful cold, itching, pricking, or pins-and-needles sensations; (ii) specific evoked pain signs, such as thermal allodynia, allodynia to light touch, or hyperalgesia; and possibly the association with (iii) focal dysautonomia signs, such as vasomotor or sudomotor disturbances, skin color change, sweating disorders.

The intrinsic reason for introducing the concept of “small fiber neuralgia” into the taxonomy of pain is to allow for the characterization of a painful neuropathic condition based on well-defined symptoms and clinical signs of small fiber involvement (see above), but without requiring concomitant objective abnormalities in small fiber complementary testing (skin biopsy, neurophysiology, autonomic tests). If these tests reveal lesion-type abnormalities in addition to the clinical picture of pain, then the diagnosis changes from “small fiber neuralgia” to “painful small fiber neuropathy”. The value of this approach is to suggest the involvement of small fibers in a painful condition, even in the absence of evidence of lesions affecting them. This corresponds to a reality where small fiber hyperactivity is clearly the cause of the pain syndrome but cannot be proven by complementary tests. In this type of situation, this lack of evidence can lead to considering the pain as “psychogenic”, even though the clinical picture is evident. For “large fiber neuralgias”, such as essential trigeminal neuralgia, it is already accepted that the diagnosis is based on the clinical picture and not on complementary tests.

How does the introduction of this new concept of “small fiber neuralgia” fit into current classifications of chronic pain used in clinical practice? The most comprehensive classification is the 11th revision of the International Classification of Diseases (International Statistical Classification of Diseases and Related Health Problems, ICD-11), which was published by the World Health Organization (WHO) and came into effect in January 2022 (21). The ICD-11 offers a comprehensive classification of chronic pain conditions, developed by a working group of the International Association for the Study of Pain (IASP), distinguishing seven main categories of chronic pain, including the diagnosis of “chronic neuropathic pain” (code MG30.5) (22, 23). The category “chronic neuropathic pain” is itself divided into five subcategories, including the diagnosis of “chronic peripheral neuropathic pain” (code MG30.51) (24). Three clinical conditions are associated with this subcategory: (i) chronic neuropathic pain after peripheral nerve injury, (ii) chronic painful polyneuropathy, (iii) chronic painful radiculopathy. Other conditions of peripheral neuropathic pain are associated with specific codes, such as chronic painful radiation-induced neuropathy (MG30.11) or postherpetic neuralgia (1E91.5).

Regarding small fiber neuropathy, the corresponding ICD-11 code is 8C0Y, included in the diagnosis of “other specified polyneuropathy” (25), a subcategory of “diseases of the nervous system (08)/disorders of nerve root, plexus or peripheral nerves/polyneuropathy”. Furthermore, the ICD-11 classification offers a specific code for small fiber autonomic neuropathy (8D88). The code 8C0Y can be used in coordination with the additional code MG30.51 related to the presence of chronic peripheral neuropathic pain. Thus, the ICD-11 classification allows for the accurate coding of “painful small fiber neuropathy”.

In contrast, the ICD-11 classification considers “neuralgia” as a “matching term” corresponding to “chronic neuropathic pain” (code MG30.5), defining only specific codes for cases of focal neuralgia involving nerve trunks: trigeminal neuralgia (8B82.0), occipital neuralgia (8A85), glossopharyngeal neuralgia (8B87), pudendal neuralgia (GA34.0Y), or even nerve roots: cervicobrachial neuralgia (SK51), sciatic neuralgia (ME84.3). Thus, more diffuse forms of neuralgia corresponding to the specific involvement of small-diameter sensory nerve fibers, without structural lesions of these fibers (“small fiber neuralgia” without “neuropathy”), could only be linked to the generic code MG30.5Z corresponding to “chronic neuropathic pain, unspecified”.

As previously mentioned, the ICD-11 classification of chronic pain is based on the IASP recommendations, which define chronic peripheral neuropathic pain as “caused by a lesion or disease of the peripheral somatosensory nervous system” (24, 26). Small fiber neuralgia, as a “disease” of the peripheral somatosensory nervous system, meets the IASP criteria for defining a condition of “peripheral neuropathic pain”. However, the IASP Specialist Interest Group on Neuropathic Pain (NeuPSIG) has proposed a grading of the diagnosis of neuropathic pain into possible, probable, and definite, with the diagnosis of “definite” neuropathic pain based on confirmation of somatosensory nervous system lesion by complementary tests (9). In the absence of such lesion and objective test abnormalities, small fiber neuralgia cannot be considered a condition of “definite” neuropathic pain according to this algorithm. This underlines the limitations of complementary tests and therefore of this algorithm for the diagnosis of neuropathic pain, as discussed in a previous publication (11).

Thus, in the absence of objective abnormalities of somatosensory nervous system lesions, small fiber neuralgia could be misdiagnosed as nociplastic pain, when it is clearly neuropathic. In fact, the differential diagnosis should be based not on complementary tests that only assess the presence of a neurological lesion, but on clinical characteristics, as illustrated by a new diagnostic algorithm for neuropathic pain proposed in a previous article (11). According to this new algorithm, the diagnosis of small fiber neuralgia should be based on patient's interview, including a report of pain descriptors specific to small fiber involvement and a distinct distribution of pain symptoms plausible for neuroanatomical involvement of these fibers. In contrast, nociplastic pain should be diagnosed in the presence of multifaceted sensory descriptors, with diffuse, non-systematized and variable pain symptoms, both spatially and temporally, associated with a significant impact of cognitive impairment (attention, concentration, or memory disorders, fatigue) and anxiety-depressive components.

In summary, the diagnosis of small fiber involvement in a chronic pain syndrome should be based exclusively on precise “qualitative clinical characteristics” concerning sensory and autonomic signs and symptoms (Figure 1). In the absence of concomitant evidence of structural lesions or loss of small nerve fibers, the proposed diagnosis should be “small fiber neuralgia”. The diagnosis of “painful small fiber neuropathy” should be reserved for the presence of concomitant lesion or loss of small nerve fibers, demonstrated by complementary tests. This will avoid any confusion between the characterization of the pain syndrome and the detection of nerve lesions, the presence of which does not necessarily imply the presence of pain.
